# *Drosophila *phosphopantothenoylcysteine synthetase is required for tissue morphogenesis during oogenesis

**DOI:** 10.1186/1756-0500-1-75

**Published:** 2008-08-29

**Authors:** Floris Bosveld, Anil Rana, Willy Lemstra, Harm H Kampinga, Ody CM Sibon

**Affiliations:** 1 Department of Cell Biology, Section of Radiation & Stress Cell Biology, University Medical Center Groningen, University of Groningen, Groningen, The Netherlands

## Abstract

**Background:**

Coenzyme A (CoA) is an essential metabolite, synthesized from vitamin B_5 _by the subsequent action of five enzymes: PANK, PPCS, PPCDC, PPAT and DPCK. Mutations in *Drosophila dPPCS *disrupt female fecundity and in this study we analyzed the female sterile phenotype of *dPPCS *mutants in detail.

**Results:**

We demonstrate that *dPPCS *is required for various processes that occur during oogenesis including chorion patterning. Our analysis demonstrates that a mutation in *dPPCS *disrupts the organization of the somatic and germ line cells, affects F-actin organization and results in abnormal PtdIns(4,5)P_2 _localization. Improper cell organization coincides with aberrant localization of the membrane molecules Gurken (Grk) and Notch, whose activities are required for specification of the follicle cells that pattern the eggshell. Mutations in *dPPCS *also induce alterations in scutellar patterning and cause wing vein abnormalities. Interestingly, mutations in *dPANK *and *dPPAT-DPCK *result in similar patterning defects.

**Conclusion:**

Together, our results demonstrate that *de novo *CoA biosynthesis is required for proper tissue morphogenesis.

## Findings

Coenzyme A (CoA), the major acyl carrier in all organisms, constitutes an essential cofactor to support cellular metabolism [1]. Synthesis of CoA occurs via a conserved route in which vitamin B_5 _is subsequently modified by five enzymes: PANK, PPCS, PPCDC, PPAT and DPCK [2-5]. Although CoA biosynthesis is well characterized in bacteria and in *in vitro *systems [6], only recently has the impact of abnormal CoA biosynthesis on animals been investigated [7-10].

### Mutations in *dPPCS *impair female fecundity and fertility

Previously, we isolated a *Drosophila dPPCS *mutant as a female sterile, neurologically impaired mutant and we demonstrated that CoA metabolism is required to maintain DNA integrity during development of the central nervous system [8]. Here, we analyzed the female sterile phenotype of a hypomorphic allele of *dPPCS *(*dPPCS*^1^) in detail. *dPPCS*^33 ^mutants (a null allele) are homozygous lethal [8], and in *dPPCS*^1/33 ^mutants, no vitellogenic egg chambers were observed. Using immunohistochemistry and confocal laser scanning microscopy (supplement) we have analyzed the defects that occur during oogenesis (see for recent reviews [11,12]).

**Figure 1:**
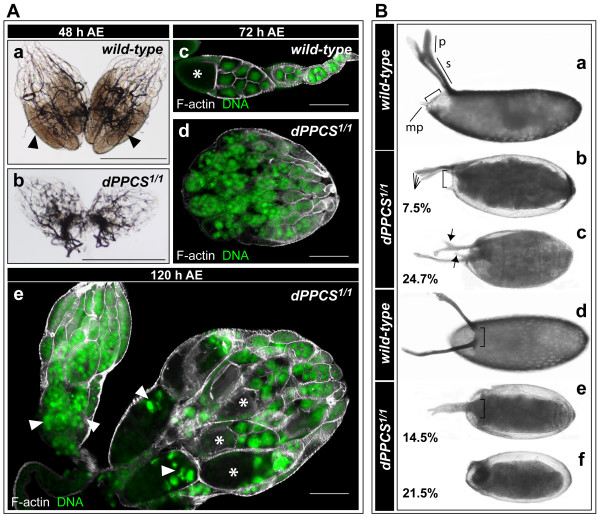
**Egg chamber development and eggshell patterning is disrupted in *dPPCS*^1/1^**. (A) Morphology of *dPPCS*^1/1 ^ovaries was analyzed and compared with wt using light microscopy. (Aa) Bright field microscopy revealed that at 48 h after eclosion (AE), Wt ovaries are well developed and contain mature eggs (arrowheads). (Ab) At 48 h after eclosion, *dPPCS*^1/1 ^ovaries were small in size compared to wt. (Ac-e) Ovaries were labeled with rhodamin-phalliodin to detect F-actin and stained with DAPI to visualize DNA. (Ac) At 72 h AE, wt ovaries contain vitellogenic egg chambers, as determined by the increased size of the oocyte compartment (asterisk). (Ad) *dPPCS*^1/1 ^ovaries remained small in size (1 entire lobe is shown) and no vitellogenic egg chambers were observed. (Ae) At 120 h AE, *dPPCS*^1/1 ^ovaries contained vitellogenic egg chambers (asterisks) and exhibited features of degenerating egg chambers (arrowheads). The 2 lobes were frequently different in size. (B) Chorion patterning was analysed in *dPPCS*^1/1^. (Ba, Bd) Wt embryos have 2 dorsal appendages. (mp = micropyle; p = paddle; s = stalk). (Bb-c, Be-f) Embryos deposited by *dPPCS*^1/1 ^mothers showed a dumpless phenotype and had a wide range of patterning defects, which were classified in 5 groups: (Bb) embryos with opercula positioned in a different angle in relation to the stalks (bracket, compare with Ba) and 4 appendages; (Bc) abnormal stalks (arrows); (Be) fused appendages (bracket, compare with Bd); and (Bf) embryos without dorsal appendages. Percentages are indicated (n = 142). The remaining 22.8% had either 2 dorsal appendages of different lengths, missing paddles, or a shift of the dorsal appendages posteriorly. Scale bars: 500 μm (Aa-b), 150 μm (Ac-e).

At 48 h after eclosion (AE), the ovaries from *dPPCS*^1/1 ^females were small compared to wild-type (wt) ovaries and mutant ovaries did not contain mature eggs (Fig. 1Aa–b). In wt, the oldest egg chambers found in newly eclosed females are at stage 7, and upon food intake, hormones are produced which trigger the egg chambers to proceed into vitellogenesis, a process whereby the oocyte accumulates nutrients and increases in size [13]. At 72 h AE, 100% (n = 35) of the wt ovaries contained vitellogenic egg chambers, while only 11% of the *dPPCS*^1/1 ^ovaries (n = 36) contained vitellogenic egg chambers (Fig. 1Ac–d). At 120 h AE, 80% of the *dPPCS*^1/1 ^ovaries (n = 26) contained vitellogenic egg chambers; however, the two lobes were frequently different in size and displayed features of degenerating egg chambers (Fig. 1Ae).

Between 144–192 h AE, *dPPCS*^1/1 ^females deposited 0.03 (± 0.02 SEM) eggs/24 h, none of which hatched (n = 142 eggs), while wt females produced 10.0 (± 1.4 SEM) eggs/24 h, of which 90% hatched (n = 1005 eggs). It has been reported that a mid-oogenesis checkpoint monitors the integrity of pre-vitellogenic egg chambers, and that activation of this checkpoint results in the removal of abnormal egg chambers [13]. A Tunnel assay was performed, which revealed that in *dPPCS*^1/1 ^ovaries at 144 h AE, prior to vitellogenesis, a 6-fold increase of ovariols containing apoptotic egg chambers was observed, compared to wt ovaries (see additional file 1). Approximately 32% of *dPPCS*^1/1 ^ovariols (n = 222) contained stage 5–7 egg chambers that displayed packaging defects (abnormal amount of germ line cells), while 4% of the wt ovariols (n = 109) contained egg chambers with packaging defects. When we expressed a *dPPCS *transgene (*P[dPPCS]*) in the *dPPCS*^1/1 ^background, 11% (n = 166) of the ovariols displayed defects, demonstrating that *dPPCS *is required for early egg chamber development. Within *dPPCS*^1/1 ^germaria, aberrant separation of the developing egg chambers by the intercyst cells likely results in production of egg chambers with abnormal interfollicular stalk cell and/or polar follicle cell formation, egg chambers with mispositioned oocytes, or egg chambers that display packaging defects (see additional file 1). Thus, the reduced fecundity of the *dPPCS*^1/1 ^females is most likely due to production of aberrant egg chambers that did not pass the mid-oogenesis checkpoint and were absorbed.

### *dPPCS *is required for F-actin remodeling during cytoplasmic dumping

**Figure 2:**
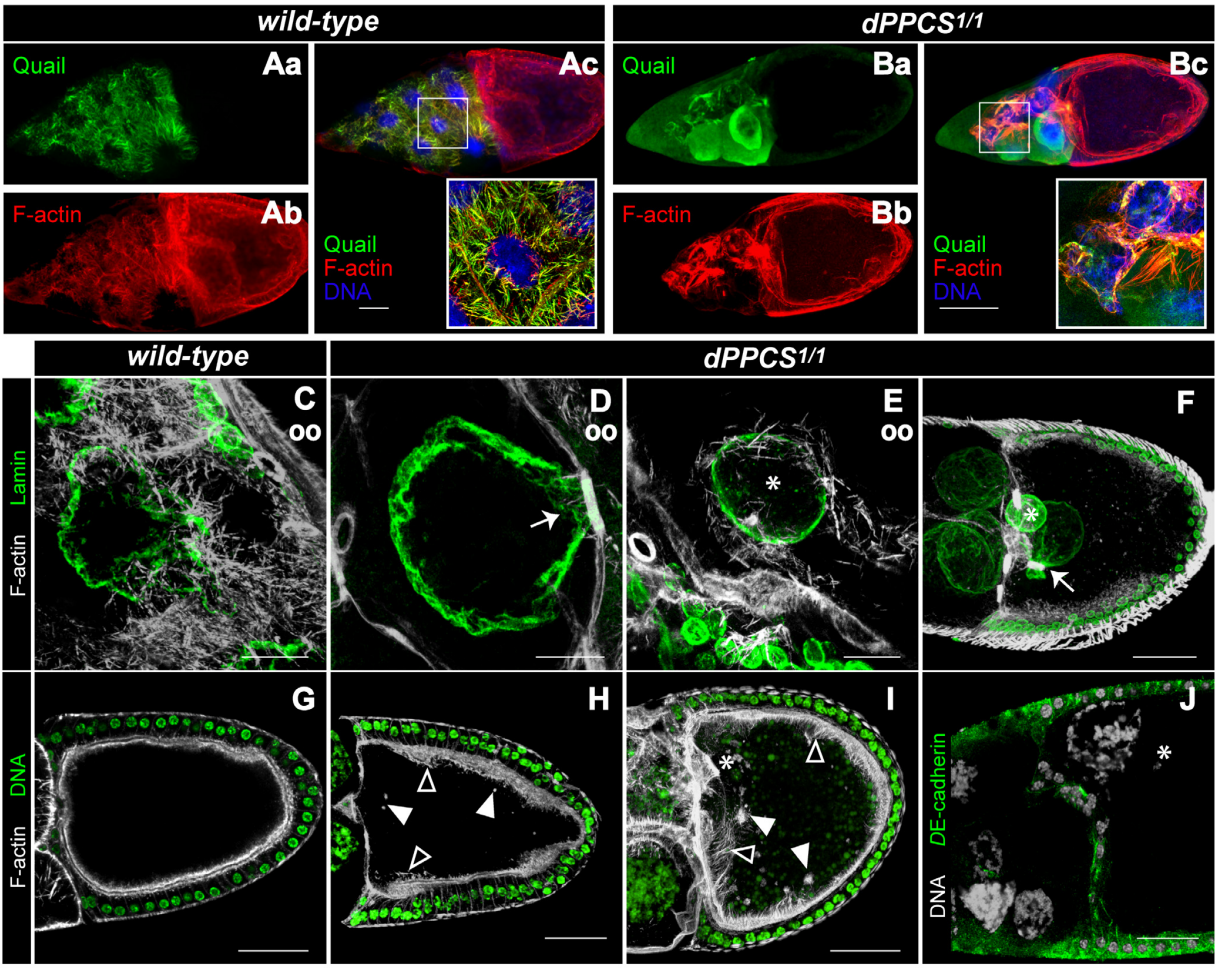
**Cytoplasmic F-actin filament assembly and dumping is disrupted in *dPPCS*^1/1 ^egg chambers**. To examine the morphology of *dPPCS*^1/1 ^mutant ovaries, rhodamin-phalloidin was used to visualize the F-actin network in combination with DAPI to stain nuclei and various other antibodies as described. (Aa-Ac) Wt nurse cells assemble an elaborate network of transverse F-actin filaments prior to cytoplasmic dumping. Labeling using antibodies against Quail revealed colocalization of Quail with F-actin filaments in wt. (Ba-Bc) The cytoplasmic F-actin network is not properly formed inside *dPPCS*^1/1 ^nurse cells, and Quail localization is diffuse inside the cytoplasm. To visualize nurse cell nuclei an antibody against lamin D_o _was used. (C) In wt ovaries, F-actin bundles anchor the nurse cell nuclei during dumping. (D) *dPPCS*^1/1 ^nurse cells fail to assemble F-actin filaments, and nurse cell nuclei trapped inside the ring canals during dumping were observed (arrows in D, F). (E) Example of a *dPPCS*^1/1 ^oocyte nucleus encapsulated by F-actin fibers. (F) In *dPPCS *mutant egg chambers, nurse cell nuclei were found inside the oocyte compartment (arrow marks a nurse cell nucleus trapped inside a ring canal). (G) During cytoplasmic dumping, a tight array of F-actin is present at the subcortical membrane of wt oocytes. (H-I) The subcortical F-actin fibers at the membrane of the *dPPCS*^1/1 ^oocyte compartment were increased in size and thickness (boxed arrowhead), and large clumps of F-actin were found within the oocyte compartment (arrowheads). (J) An antibody against *D*E-cadherin was used to visualize centripetal migrating follicle cells because these cells express high levels of *D*E-cadherin. In *dPPCS *mutants, migration of these cells occurred normally, but nurse cells are observed within the oocyte compartment after these cells finished their migration. An example of a nurse cell nucleus in the dorsoanterior corner of the *dPPCS*^1/1 ^oocyte compartment is shown. Asterisks mark the oocyte nuclei. (oo) oocyte compartment. Scale bars: 100 μm (A-B), 20 μm (C-E), 50 μm (F-J).

In addition to impaired fecundity, 80% of the eggs deposited by *dPPCS*^1/1 ^females displayed a dumpless phenotype [14] and a wide array of chorion patterning defects (Fig. 1B). Since patterning defects can arise from aberrant actin fiber formation within the nurse cells [15,16], we analyzed actin formation during cytoplasmic dumping. In stage 10 wt egg chambers, an elaborate network of F-actin bundles is assembled inside the nurse cells which is a prelude to cytoplasmic dumping. These bundles anchor the nurse cell nuclei to prevent them from entering the oocyte when the remaining nurse cell material is actively squeezed into the oocyte [17]. Assembly of this F-actin network requires the Quail protein, which colocalizes with the F-actin fibers (Fig. 2A) [16,18]. In *dPPCS*^1/1 ^egg chambers, assembly of the cytoplasmic F-actin fibers was disrupted, and the Quail protein failed to associate with the F-actin bundles and remained diffuse throughout the nurse cell cytoplasm (Fig. 2B). As a result of aberrant F-actin assembly, nurse cell nuclei were trapped inside ring canals during dumping (Fig. 2D, Table 1). Interestingly, we also found oocyte nuclei that were encapsulated by bundles of actin (Fig. 2E, Table 1). Furthermore, large actin fibers were assembled at the cortical membrane of the oocyte, and the follicular epithelium of the oocytes was frequently disorganized (Fig. 2H–I). Mutant oocytes also contained large clumps of F-actin (Fig. 2H–I, Table 1) and we frequently found nurse cell nuclei inside the oocyte compartment (Fig. 2F, J, Table 1).

**Table 1:** Mutations in *dPPCS *affect egg chamber development, stage 10–11 F-actin organization & cytoplasmic dumping

	% of egg chambers		
	
	*wild-type*	*dPPCS*^1/1^	*P[dPPCS];dPPCS*^1/1^
Nurse cells trapped inside the oocyte	0.0 (n = 100)	20.9 (n = 67)	1.8 (n = 56)
F-actin clumps in ooplasm	3.0 (n = 100)	50.1 (n = 53)	7.0 (n = 57)
Aberrant F-actin in nurse cells	0.0 (n = 100)	92.2 (n = 51)	29.5 (n = 61)
Nurse cells plugging ring canals	0.0 (n = 100)	71.0 (n = 62)	15.5 (n = 58)
Oocytes with disorganized subcortical F-actin	0.0 (n = 100)	43.7 (n = 55)	0.0 (n = 53)
Oocyte nuclei with F-actin fibers	0.0 (n = 100)	18.8 (n = 48)	0.0 (n = 46)

Nurse cells were stained with DAPI to detect DNA and labeled with rhodamin-phalloidin to visualize the F-actin network.

*P[dPPCS] *is a FLAG-tagged dPPCS cDNA under control of a ubiquitin promoter.

**Figure 3:**
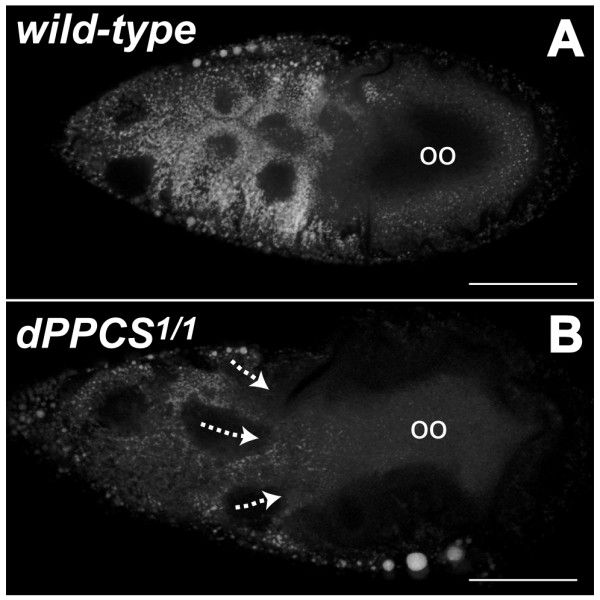
**Synthesis and transport of neutral lipids is hampered in *dPPCS *mutant egg chambers**. Freshly dissected wt and *dPPCS*^1/1 ^ovaries were stained with Nile red to visualize neutral lipids and dissected ovaries were directly analyzed by CLSM. Images represent single confocal scans. (A) Wt nurse cells produce high levels of neutral lipids, which are transported towards the oocyte, and are uniformly accumulated near the oocyte membrane. (B) *dPPCS*^1/1 ^nurse cells produced less neutral lipids compared to wt nurse cells. No uniformly accumulated lipids were observed within the mutant oocyte compartment compared to wt. (oo) oocyte compartment. Scale bars: 100 μm.

We stained freshly dissected ovaries with Nile red, which has fluorescent properties in the presence of triacylglycerol and sterol esters [19], to determine if neutral lipid synthesis and transport of these lipids to the oocyte was disrupted during cytoplasmic dumping. In wt, synthesis of these neutral lipids increases in the germ line and somatic cells when egg chambers proceed into late stage oogenesis, and these neutral lipids are transported to the oocyte where they accumulate uniformly near the oocyte membrane (Fig. 3A). In *dPPCS*^1/1 ^egg chambers, neutral lipid synthesis was reduced compared to wt, suggesting that the synthesis of neutral lipids is affected in *dPPCS *mutants (Fig. 3B). Furthermore, accumulation inside the oocyte of these lipids appeared abnormal compared to wt ovaries (compare Fig. 3A and 3B).

**Figure 4:**
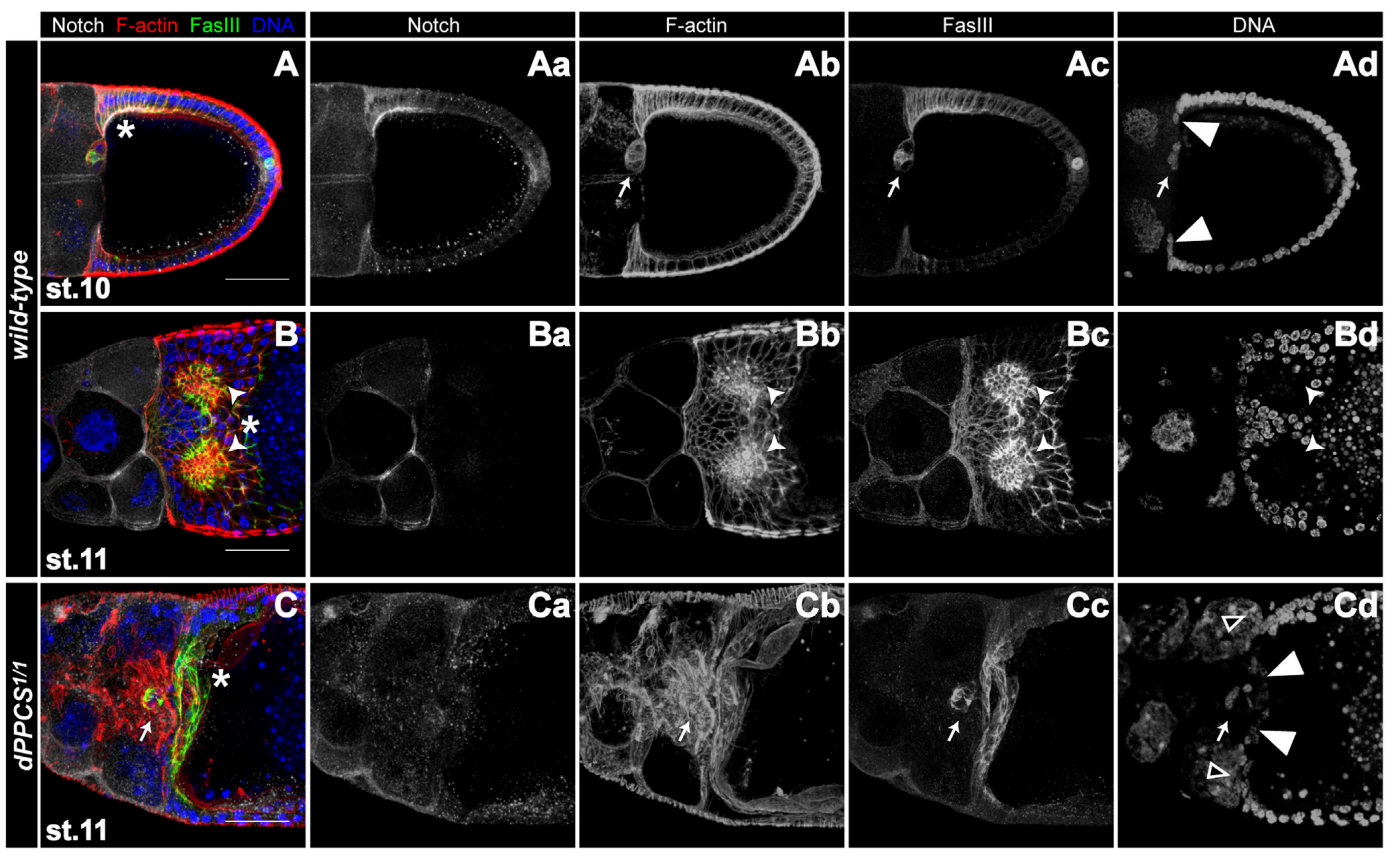
**A mutation in *dPPCS *affect follicle cell migration and patterning**. *dPPCS*^1/1 ^and wt egg chambers were stained with rhodamin falloidin (to visualize F-actin) and DAPI to stain nuclei and labeled with antibodies against FasIII and Notch to analyze follicle cell migration and patterning. (Aa-Ad) Wt stage 10 egg chamber. During stages 8–10, the border cells migrate through the nurse cell compartment towards the anterior end of the oocyte. When the border cells (arrow) reach the oocyte (stage 10) and the centripetal follicle cells start migrating (arrowheads), FasIII is expressed in the follicle cells of the dorsoanterior corner. At this stage, Notch is expressed at the membranes of the follicle cells of the dorsoanterior corner, where it is required for the specification of the dorsal appendage producing cells. (Ba-Bd) During stage 11, after the centripetal cells finished their migration, two patches of follicle cells can be found at the dorsoanterior corner of the epithelium of wt egg chambers (arrowheads). These follicle cells express high levels of FasIII and will initiate the production of the dorsal appendages. At this stage, Notch expression is restricted to the nurse cell membranes. (Ca-Cd) *dPPCS*^1/1 ^egg chamber at stage 11. The centripetal follicle cells finished migration (arrowheads), but the border cells (arrow) failed to reach the centripetal follicle cells, while follicle cells of the dorsoanterior corner were already expressing FasIII. The border cell cluster is surrounded by an elaborate network of F-actin. Notch localization is not restricted to the nurse cell membranes and shows a more diffuse pattern. Boxed arrowheads point to two nurse cell nuclei that seem to contact (push against) the centripetal follicle cells. Asterisks mark the position of the oocyte. Scale bars: 50 μm.

Next, we investigated whether a mutation in *dPPCS*^1/1 ^affects cell migration events due to defective F-actin organization. During stages 8–10, the border cells, which include the anterior polar cells and part of the main body epithelium, migrate through the nurse cell compartment towards the anterior end of the oocyte [20]. In wt, when the border cells reach the oocyte and the centripetal follicle cells start migrating, Fasciclin III (FasIII) is expressed in the follicle cells of the dorsoanterior corner (Fig 4A). After centripetal follicle cells finished their migration, FasIII expressing cells form two distinct cell populations at the dorsoanterior surface of the oocyte. Here, formation of the dorsal appendages is initiated (Fig 4B) [14]. In *dPPCS*^1/1 ^egg chambers, centripetal migration was finished before the border cells reached the anterior of the oocyte (Fig 4C), indicating that these two cell migration events are not properly synchronized. Together, these data demonstrate that *dPPCS *is required for F-actin organization and cell migration events during oogenesis.

### Grk and Notch localization is disrupted in *dPPCS^1/1^*egg chambers

We hypothesized that disorganized tissue integrity may also affect the signaling routes required for specification of follicle cells that pattern the chorion. To investigate this, we stained ovaries with antibodies against Notch and Grk, which both are required for specification of the follicle cell populations that pattern the eggshell [14,21]. Although we cannot conclude that Grk or Notch signaling was disrupted in *dPPCS*^1/1 ^ovaries, the localization of both proteins was frequently impaired compared to wt ovaries. In wt egg chambers, when the border cells reach the centripetal follicle cells, Notch is highly expressed at the dorsoanterior corner, where it is required for the specification of the dorsal appendage producing cells, while Notch expression is restricted to the nurse cell membranes during cytoplasmic dumping (Fig. 4Ba, see additional file 1) [22]. In *dPPCS*^1/1 ^stage 11 egg chambers, Notch localization was more diffuse throughout the nurse cells and not restricted to the membranes (Fig. 4Ca). Notch localization was also severely affected during late stage oogenesis (see additional file 1) and FasIII staining revealed that the dorsal appendage/operculum forming follicle cells were not properly organized (see additional file 1).

In wt stage 9–10 egg chambers, Grk is localized at the dorsoanterior corner of the oocyte compartment. Although in *dPPCS*^1/1 ^egg chambers, Grk was present at the dorsoanterior corner, the distribution of the protein was frequently impaired in stage 8–9 egg chambers (see additional file 1) and progressively worsened when egg chambers proceeded into later stages of oogenesis (see additional file 1).

These findings imply that *dPPCS *is not required for cell specification or signaling per se, but merely required for cell organization and morphology. This is supported by the finding that aberrant intercyst cell migration/organization likely underlies the observed packaging and follicle cell specification defects during early oogenesis (see additional file 1).

### Membrane localization of PtdIns(4,5)P_2 _is impaired in *dPPCS*^1/1^

The levels of phospholipids are reduced in *dPPCS *mutant flies, indicating a general defect in phospholipid biosynthesis [8]. Therefore, it is plausible to assume that phosphatidylinositol (PtdIns) production, the precursor for all phosphoinositides [23], is also reduced. Although levels and localization of PtdIns have not been determined during *Drosophila *oogenesis, it is generally accepted that actin remodeling processes depend on PtdIns signaling [24].

**Figure 5:**
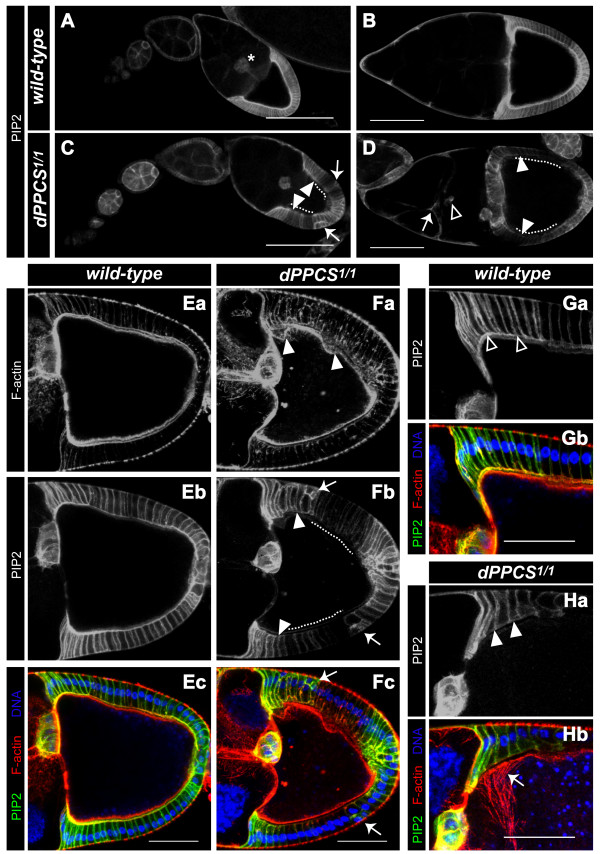
**PtdIns(4,5)P_2 _localization and expression is affected in *dPPCS*^1/1^**. We overexpressed a PLCδ-PH-GFP fusion protein [25] under the control of a ubiquitously expressed Act5C-GAL4 driver to analyze PtdIns(4,5)P_2 _localization and expression in a wt (A,B,E,G) and *dPPCS*^1/1 ^(C,D,F,H) background. (A,B) During wt oogenesis, the PLCδ-PH-GFP fusion protein is present at the border cells (asterisk) and the apical membranes of the follicle cells that encapsulate the oocyte compartment, while the nurse cell membranes do not accumulate the fusion protein. (C,D) In *dPPCS*^1/1 ^egg chambers, the PLCδ-PH-GFP fusion protein is not present at the apical membranes of the follicle cells that encapsulate the oocyte (arrowheads) and large patches of follicle cells are not labeled (dashed lines). In mutant ovaries, the follicular epithelium is sometimes disrupted (arrows in C), and the nurse cell membranes accumulate patches of high levels of the PLCδ-PH-GFP fusion protein (arrow in D). The PLCδ-PH-GFP fusion protein was also frequently detected at membranes of cells that are (based on their localization) most likely border cells (boxed arrowhead in D). (E-H) For further analysis, F-actin organization was analyzed in combination with localization of the PLCδ-PH-GFP fusion protein. (Ea-Ec) In wt egg chambers, the oocyte cortex is in close contact with the apical membranes of the follicle cells, which accumulate the PLCδ-PH-GFP fusion protein. (F) In *dPPCS*^1/1 ^egg chambers, the oocyte cortex is disrupted (arrowheads in Fa) and the follicle cells do not accumulate the PLCδ-PH-GFP fusion protein (arrowheads and dashed lines in Fb). Arrows point to defects in cell organization of the follicular epithelium. (G) Higher magnification of a wt egg chamber, showing that the PLCδ-PH-GFP fusion protein accumulates at the apical membranes of follicle cells in close contact with the oocyte cortex (boxed arrowheads). (H) Higher magnification of a *dPPCS*^1/1 ^egg chamber, showing that the apical membranes of follicle cells in close contact with the oocyte cortex do not accumulate the PLCδ-PH-GFP fusion protein (arrowheads in Ha). This coincides with impaired oocyte cortex morphology and aberrant F-actin nucleation (arrow in Hb). DAPI was used to visualize DNA. Scale bars: 150 μm (A,C), 100 μm (B,D), 50 μm (E-H).

To investigate whether PtdIns signaling was affected in *dPPCS *mutant ovaries, we expressed a PLCδ-PH-GFP fusion protein, which is able to bind to PtdIns(4,5)P_2 _[25]. We used an Act5C-GAL4 driver to analyze PLCδ-PH-GFP expression and thus PtdIns(4,5)P_2 _localization in all cells. During wt cytoplasmic dumping, PtdIns(4,5)P_2 _is abundant at the cell membranes of the border cells and the apical membranes of the follicle cells that encapsulate the oocyte, while low levels of PtdIns(4,5)P_2 _can be detected at the nurse cell membranes (Fig. 5A,B,E). In contrast, PtdIns(4,5)P_2 _localization at the apical membranes of the follicle cells that encapsulate the oocyte was hardly detectable or absent in *dPPCS*^1/1 ^egg chambers (Fig. 5C,D,F,H). Moreover, large patches of follicle cells that encapsulate the oocyte did not accumulate PtdIns(4,5)P_2 _at their membranes (Fig. 5C,D,F). Because aberrant apical localization of PtdIns(4,5)P_2 _at the follicle cell membranes coincides with impaired oocyte cortex integrity and abnormal F-actin organization (Fig. 5H), this suggests that altered PtdIns(4,5)P_2 _signaling could underlie the F-actin defects in *dPPCS*^1/1 ^egg chambers.

Although the F-actin/PtdIns(4,5)P_2 _connection should be investigated in more detail, we propose that F-actin remodeling within the *Drosophila *ovary likely depends on PtdIns(4,5)P_2 _signaling and that this lipid derived signaling route is disrupted in *dPPCS*^1/1^. Abnormal cytoskeletal organization in *dPPCS*^1/1 ^disrupts the overall shape of all membranous structures and the organization of the cells during morphogenesis. Disorganized tissue integrity could affect Notch and Grk localization and possibly signaling, which is required for specification of the follicle cells that pattern the eggshell, and causes severe chorion patterning defects.

### *dPPCS *is required for patterning of various tissues

Next, we wondered whether dPPCS is also required for morphogenesis of other tissues. Hereto, we closely investigated *dPPCS*^1/1 ^flies for other morphological abnormalities. A stereotypical pattern of four scutellars exists on the dorsal surface of the wt scutellum, and *dPPCS *mutants displayed ectopic formation of scutellars (see additional file 1). Furthermore, *dPPCS*^1/1 ^flies also developed ectopic wing veins (see additional file 1). Mutants initiated longitudinal vein formation between L3–L4 and L4–L5. These results show that *dPPCS *is required for morphogenesis of various tissues during *Drosophila *development.

### Mutations in *de novo *CoA synthesis disrupt morphogenesis

Next, we investigated whether mutations in other CoA biosynthesis enzymes give rise to similar defects. Indeed, mutations in *dPANK/fumble *and the bifunctional enzyme *dPPAT-DPCK *result in similar characteristics compared to the *dPPCS *mutant phenotype. *dPANK/fumble *and *dPPAT-DPCK *mutant females have poorly developed ovaries, have fecundity defects, produce eggs that exhibit polarity defects, synthesize abnormal neutral lipids (droplets), and these mutants display scutellar and wing vein patterning defects (see additional file 1). As in *dPPCS*^1/1^, a mutation in *dPPAT-DPCK *disrupts actin localization and results in plugging of the ring canals by nurse cell nuclei during dumping (see additional file 1). *dPANK/fumble *mutants produce small ball-shaped eggs, which are typically due to a loss of actin regulatory elements that control the polarized arrangement of F-actin fibers at the basal cortex of follicle cells required to establish planar cell polarity [11]. These findings imply that impaired CoA synthesis in general disrupts morphogenesis, possibly due to aberrant F-actin organization. Because the biosynthesis route towards the production of CoA is conserved amongst species it would be interesting to explore the significance of CoA during processes that involve actin/PtdIns dynamics such as chemotaxis, axon growth cone guidance, endocytosis/exocytosis, cell division or actin dependent chromatin remodeling.

## Competing interests

The authors declare that they have no competing interests.

## Authors' contributions

FB, AR and OCMS conceived and designed the experiments. FB, AR and WL performed the experiments. FB, OCMS and HHK analyzed the data. FB and OCMS wrote the manuscript.

## Supplementary Material

Additional file 1 dPPCS, dPANK/fumble and dPPAT-DCPK mutants show comparable defects during oogenesis, and abnormal vein and scutellar patterning. The data provided show additional morphological information concerning defective egg chamber development and abnormal vein and scutellar patterning in dPPCS, dPANK/fumble and dPPAT-DCPK mutants. Click here for file
