# Supplementary material for: Drosophila phosphopantothenoylcysteine synthetase is required for tissue morphogenesis during oogenesis
Source: BMC Res Notes. 2008 Aug 29;1:75. doi: 10.1186/1756-0500-1-75 (PMC2542404; doi:10.1186/1756-0500-1-75)
Supplement: Additional file 1 — dPPCS, dPANK/fumble and dPPAT-DCPK mutants show comparable defects during oogenesis, and abnormal vein and scutellar patterning. The data provided show additional morphological information concerning defective egg chamber development and abnormal vein and scutellar patterning in dPPCS, dPANK/fumble and dPPAT-DCPK mutants. [file 1756-0500-1-75-S1.pdf]

## Experimental procedures

***Drosophila* stocks.** Fly stocks were maintained at 22$^{o}$C according to standard protocols. For wt preparations, $y^1w^{1118}$ was used. *dPANK$^1$* (*fumble*), *dPPAT-DPCK$^{43}$*, *dPPCS$^1$*, *dPPCS$^{33}$* and *P[dPPCS]* are previously described [1,2]. The *UAS-PLCδ-PH-GFP* line was a gift from L. Cooley and A. Wodarz and the *Act5C-GAL4* line was obtained from the Bloomington Stock Centre (Indiana University, USA).

**Immunohistochemistry.** Dissection, fixation and immunolabeling of ovaries was performed as previously described [3]. Primary antibodies used included concentrated supernatants obtained from the Developmental Studies Hybridoma Bank (Iowa, USA), mouse anti-lamin Do (ADL84.12, 1:5) developed by P.A. Fisher, mouse anti-fasciclin III (7G10, 1:5) developed by C. Goodman, mouse anti-Notch (C17.9C6, 1:5) developed by S. Artavanis-Tsakonas, mouse anti-quail (6B9, 1:5) developed by L. Cooley, mouse anti-DE-cadherin, (DCAD2, 1:5) developed by T. Uemura, mouse anti-gurken (1D12, 1:5) developed by T. Schupbach, mouse anti-orb (6H4, IgG2a, 1:5) developed by P. Schedl, mouse anti-armadillo (N27A1, IgG2a, 1:5) developed by E. Wieschaus, and mouse anti-Histone H3 pS10 (1:100, Cell Signaling). Rabbit anti-Vasa (1:50) was a kind gift of P. Lasko. Secondary antibodies included Cy3-conjugated goat anti-mouse (1:200, Jackson ImmunoResearch), FITC-conjugated goat anti-mouse(1:200,JacksonImmunoResearch),Cy5-conjugatedgoatanti-mouse1:200,JacksonImmunoResearch) and Alexa 647 goat anti-mouse IgG2a (1:200, Molecular probes). Ovaries were stained with 20 U/ml rhodamin-phalloidin (Molecular Probes) and 0.2 μg/ml DAPI (Sigma) to visualize F-actin and DNA, respectively. Apoptosis was measured in 6-d-old flies that were kept for 3 days in vials containing yeast paste. The TUNEL cell death assay was performed following the ApopTag Fluorescien *In Situ* Apoptosis Detection Kit (Chemicon). Ovaries were fixed in devitellizing buffer/heptane [3] (1:6 per volume) and pretreated with proteinase K (20 ug/ml in PBS + 0.1% Tween-20 ) for 15 min at room temperature. After labeling and washing, ovaries were mounted in citifluor (Agar Scientific), and analyzed by confocal laser scanning microscopy (CLSM) (Leica TCS SP2 DM RXE). Images represent maximal projections (unless otherwise noted) of a z-stack (0.5-1 μm/scan). Images were processed using Leica software and Paint Shop Pro. For Nile red (Sigma) staining 6-d-old flies were kept for 3 days in vials containing yeast paste. Ovaries were dissected and stained with 100 ng/ml Nile red solution in PBS for 5 min. Ovaries were washed 3 times for 10 min with PBS, mounted in citifluor and directly analyzed by CLSM.

**Assessment of fecundity and morphological analyses.** For studies of fecundity, 10 groups of 5 virgin females and 5 males were crossed for 5 days in vials containing yeast paste. Flies were transferred to fresh vials without yeast and transferred to fresh vials every 24 h. The average number of eggs deposited/24 h was calculated from 3 replicates of each group. To assay embryonic viability, embryos were collected (0-6 h) on apple juice plates containing yeast paste, counted, and the hatch rate was determined by visual inspection of the egg cases two days after egg laying. Inspection of chorion morphology was carried out using LM. Images were captured with an Olympus BX50 light microscope. To analyze ovary morphology, virgin females were placed in vials containing yeast paste and ovaries were dissected 48 h, 72 h and 120 h AE. Ovaries dissected 48 h AE were directly analyzed by light microscopy (LM) (Olympus BX50), while ovaries dissected 72 h and 120 h AE were fixed, labeled and inspected by CLSM. Assessment of wing venation and sensory organ patterning was performed by LM.

## Supplementary references

1. Afshar K, Gonczy P, DiNardo S, Wasserman SA: **fumble encodes a pantothenate kinase homolog required for proper mitosis and meiosis in Drosophila melanogaster.** Genetics 2001, **157:** 1267-1276.
2. Bosveld F, Rana A, van der Wouden PE, Lemstra W, Ritsema M, Kampinga HH et al.: **De novo CoA biosynthesis is required to maintain DNA integrity during development of the Drosophila nervous system.** Hum Mol Genet 2008 **17:**2058-2069
3. Verheyen E, Cooley L: **Looking at oogenesis.** Methods Cell Biol 1994, **44:** 545-561.
4. Bai J, Montell D: **Eyes absent, a key repressor of polar cell fate during Drosophila oogenesis.** Development 2002, **129:** 5377-5388.

5. Margolis J, Spradling A: **Identification and behavior of epithelial stem cells in the Drosophila ovary.** Development 1995, **121:** 3797-3807.
6. Tworoger M, Larkin MK, Bryant Z, Ruohola-Baker H: **Mosaic analysis in the drosophila ovary reveals a common hedgehog-inducible precursor stage for stalk and polar cells.** Genetics 1999, **151:** 739-748.
7. Ruohola H, Bremer KA, Baker D, Swedlow JR, Jan LY, Jan YN: **Role of neurogenic genes in establishment of follicle cell fate and oocyte polarity during oogenesis in Drosophila.** Cell 1991, **66:** 433-449.
8. Bilder D, Li M, Perrimon N: **Cooperative regulation of cell polarity and growth by Drosophila tumor suppressors.** Science 2000, **289:** 113-116.
9. Grammont M, Irvine KD: **fringe and Notch specify polar cell fate during Drosophila oogenesis.** Development 2001, **128:** 2243-2253.
10. Grammont M, Irvine KD: **Organizer activity of the polar cells during Drosophila oogenesis.** Development 2002, **129:** 5131-5140.
11. Lopez-Schier H, St Johnston D: **Delta signaling from the germ line controls the proliferation and differentiation of the somatic follicle cells during Drosophila oogenesis.** Genes Dev 2001, **15:** 1393-1405.
12. Torres IL, Lopez-Schier H, St Johnston D: **A Notch/Delta-dependent relay mechanism establishes anterior-posterior polarity in Drosophila.** Dev Cell 2003, **5:** 547-558.
13. Brown EH, King RC: **Studies on the events resulting in the formation of an egg chamber in Drosophila melanogaster.** Growth 1964, **28:** 41-81.
14. Hawkins NC, Thorpe J, Schupbach T: **Encore, a gene required for the regulation of germ line mitosis and oocyte differentiation during Drosophila oogenesis.** Development 1996, **122:** 281-290.
15. O'Reilly AM, Ballew AC, Miyazawa B, Stocker H, Hafen E, Simon MA: **Csk differentially regulates Src64 during distinct morphological events in Drosophila germ cells.** Development 2006, **133:** 2627-2638.
16. Lantz V, Chang JS, Horabin JI, Bopp D, Schedl P: **The Drosophila orb RNA-binding protein is required for the formation of the egg chamber and establishment of polarity.** Genes Dev 1994, **8:** 598-613.
17. Hay B, Jan LY, Jan YN: **Localization of vasa, a component of Drosophila polar granules, in maternal-effect mutants that alter embryonic anteroposterior polarity.** Development 1990, **109:** 425-433.
18. Jackson SM, Blochlinger K: **cut interacts with Notch and protein kinase A to regulate egg chamber formation and to maintain germline cyst integrity during Drosophila oogenesis.** Development 1997, **124:** 3663-3672.
19. Zhang Y, Kalderon D: **Regulation of cell proliferation and patterning in Drosophila oogenesis by Hedgehog signaling.** Development 2000, **127:** 2165-2176.
20. Oh J, Steward R: **Bicaudal-D is essential for egg chamber formation and cytoskeletal organization in drosophila oogenesis.** Dev Biol 2001, **232:** 91-104.
21. Besse F, Busson D, Pret AM: **Fused-dependent Hedgehog signal transduction is required for somatic cell differentiation during Drosophila egg chamber formation.** Development 2002, **129:** 4111-4124.
22. Goode S, Melnick M, Chou TB, Perrimon N: **The neurogenic genes egghead and brainiac define a novel signaling pathway essential for epithelial morphogenesis during Drosophila oogenesis.** Development 1996, **122:** 3863-3879.
23. Horne-Badovinac S, Bilder D: **Mass transit: epithelial morphogenesis in the Drosophila egg chamber.** Dev Dyn 2005, **232:** 559-574.
24. Deng WM, Althauser C, Ruohola-Baker H: **Notch-Delta signaling induces a transition from mitotic cell cycle to endocycle in Drosophila follicle cells.** Development 2001, **128:** 4737-4746.
25. Dej KJ, Spradling AC: **The endocycle controls nurse cell polytene chromosome structure during Drosophila oogenesis.** Development 1999, **126:** 293-303.

26. King R.C.. **Ovarian development in Drosophila melanogaster.**, Academic Press, New York, 1970.
27. Patel NH, Snow PM, Goodman CS: **Characterization and cloning of fasciclin III: a glycoprotein expressed on a subset of neurons and axon pathways in Drosophila.** Cell 1987, **48:** 975-988.
28. Peifer M, Orsulic S, Sweeton D, Wieschaus E: **A role for the Drosophila segment polarity gene armadillo in cell adhesion and cytoskeletal integrity during oogenesis.** Development 1993, **118:** 1191-1207.
29. Godt D, Tepass U: **Drosophila oocyte localization is mediated by differential cadherin-based adhesion.** Nature 1998, **395:** 387-391.
30. Gonzalez-Reyes A, St Johnston D: **The Drosophila AP axis is polarised by the cadherin-mediated positioning of the oocyte.** Development 1998, **125:** 3635-3644.
31. Mahowald AP, Strassheim JM: **Intercellular migration of centrioles in the germarium of Drosophila melanogaster. An electron microscopic study**. J Cell Biol 1970, **45:** 306-320.
32. Lopez-Schier H: **The polarisation of the anteroposterior axis in Drosophila.** Bioessays 2003, **25:** 781-791.
33. Xu T, Caron LA, Fehon RG, Artavanis-Tsakonas S: **The involvement of the Notch locus in Drosophila oogenesis.** Development 1992, **115:** 913-922.
34. D**uffy JB, Harrison DA, Perrimon N:** Identifying loci required for follicular patterning using directed mosaics. Development 1998, **125:** 2263-2271.
35. Bateman J, Reddy RS, Saito H, Van Vactor D: **The receptor tyrosine phosphatase Dlar and integrins organize actin filaments in the Drosophila follicular epithelium.** Curr Biol 2001, **11:** 1317-1327.
36. Frydman HM, Spradling AC: **The receptor-like tyrosine phosphatase lar is required for epithelial planar polarity and for axis determination within drosophila ovarianfollicles.** Development 2001, **128:** 3209-3220.
37. Gutzeit HO, Eberhardt W, Gratwohl E: **Laminin and basement membrane-associated microfilaments in wild-type and mutant Drosophila ovarianfollicles.** J Cell Sci 1991, **100 ( Pt 4):** 781-788.
38. Deng WM, Schneider M, Frock R, Castillejo-Lopez C, Gaman EA, Baumgartner S et al.: **Dystroglycan is required for polarizing the epithelial cells and the oocyte in Drosophila.** Development 2003, **130:** 173-184.
39. Deng WM, Ruohola-Baker H: **Laminin A is required for follicle cell-oocyte signaling that leads to establishment of the anterior-posterior axis in Drosophila.** Curr Biol 2000, **10:** 683-686.
40. Mahajan-Miklos S, Cooley L: **The villin-like protein encoded by the Drosophila quail gene is required for actin bundle assembly during oogenesis.** Cell 1994, **78:** 291-301.
41. Gutzeit HO: **The microfilament pattern in the somatic follicle cells of mid-vitellogenic ovarianfollicles of Drosophila.** Eur J Cell Biol 1990, **53:** 349-356.
42. Adler PN: **Planar signaling and morphogenesis in Drosophila.** Dev Cell 2002, **2:** 525-535.
43. Vereshchagina N, Wilson C: **Cytoplasmic activated protein kinase Akt regulates lipid-droplet accumulation in Drosophila nurse cells.** Development 2006, **133:** 4731-4735.

**Packaging defect in *dPPCS*$^{1/1}$ egg chambers are likely due to impaired intercyst cell behavior**
**Supplementary Fig. S1** shows that *dPPCS*$^{1/1}$ germaria display: **1)** incomplete follicle cell migration; **2)** fusion of cysts; **3)** aberrant formation of the interfollicular stalk that separates neighboring egg chambers; and **4)** cysts in region 2b of the germarium that do not show the characteristic lens-shape, indicative of aberrant encapsulation of cysts. Furthermore, stage 3-6 *dPPCS*$^{1/1}$ egg chambers with mispositioned oocytes show ectopic polar follicle cells and egg chambers are frequently present that are not separated by interfollicular stalk cells. In addition, egg chambers are present that are separated by elongated stalks containing undifferentiated follicle cells. Proper formation of these stalk cells is essential to ensure packaging of the egg chambers prior to budding from the germarium. Because the polar follicle and the stalk cell populations are derived from the intercyst cells [4-6], it is possible that packaging defects in *dPPCS*$^{1/1}$ are due to aberrant intercyst cell specification and/or organization [7-12]. Aberrant follicle cell specification/organization is supported by the finding that in *dPPCS*$^{1/1}$ mutants, FasIII localization is also detected in stage 7 egg chambers, indicating that differentiation of the cuboidal follicle cells that encapsulate the egg chambers is sometimes disrupted. Moreover, *dPPCS*$^{1/1}$ egg chambers are present in which the follicle cells accumulate in a bilayer at the posterior of the oocyte and Notch is frequently abnormally localized in mutant germaria. In *dPPCS*$^{1/1}$ mutants, egg chambers with supernumerary nurse cells and oocytes are present and the ratio of nurse cells to oocytes is not always 15:1. In addition, the mispositioned oocyte(s) (n >100) in *dPPCS*$^{1/1}$ egg chambers do not have more than the normal amount of 4 ring canals [13], suggesting that supernumerary cells are not due to extra cell divisions with incomplete cytokinesis [14]. Similarly, supernumerary cells are also not the result of extra cell division with complete cytokinesis, because extra germ cells are accompanied by extra ring canals [15]. *dPPCS*$^{1/1}$ oocytes accumulate normal amounts of the Orb protein [16], and the germ line cells accumulate normal amounts of the Vasa protein [17]. Therefore, the observed supernumerary cells are not due to disrupted germ cell identity or oocyte specification. Finally, no defects in follicle cell proliferation are present (no major gaps in the follicular epithelia we detected); therefore, the supernumerary nurse cells and oocytes are not a result of abnormal follicle cell proliferation [18-21]. Taken together, these analyses indicate that packaging defects in *dPPCS*$^{1/1}$ females may result from abnormal cyst encapsulation and abnormal budding due to aberrant intercyst cell behavior and organization. Because we observed abnormal cyst development from region 2b onwards, we believe that *dPPCS* is required for normal encapsulation of the cyst by the intercyst cells [22]. Aberrant migratory behaviour or organization of the intercyst cells likely induces mislocalization of the Armadillo (Arm), DE-cadherin (DE-cad) and Notch expressing follicle cells, which, in turn, disrupts differentiation of the stalk cells, the polar follicle cells and the cuboidal follicle cells. Concomitantly, cyst encapsulation, anteroposterior axis formation (germ line cell rearrangement) and budding of follicles is disrupted. Because the correct positioning of the germ line cells and the follicle cells in the germarium is largely driven by cytoskeletal rearrangements and changes in cell adhesion [23], it is also possible that a mutation in *dPPCS* affects cell organization/ migration by disrupting cytoskeletal remodeling due to changes in PtdIns homeostasis within the germarium.

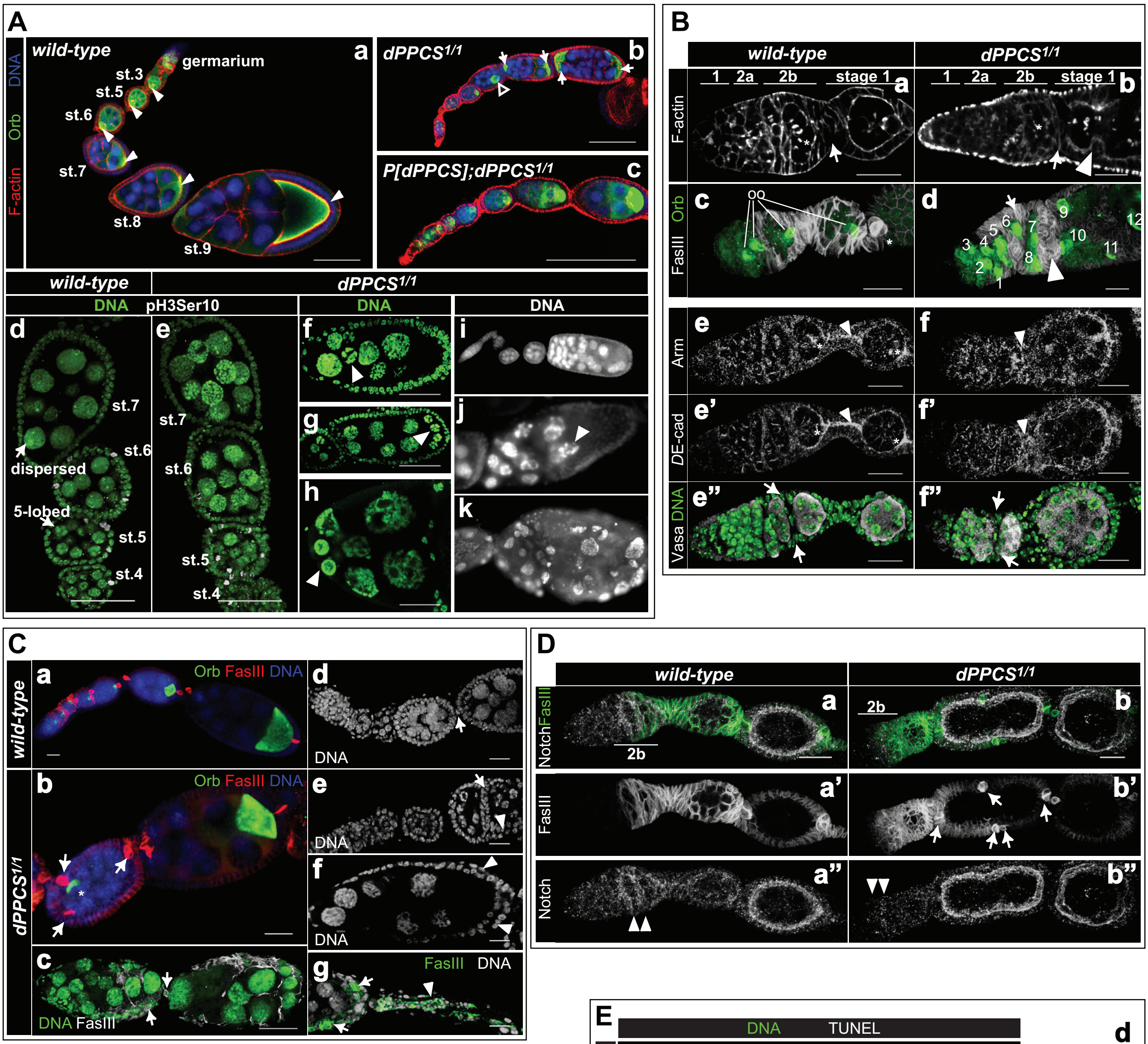


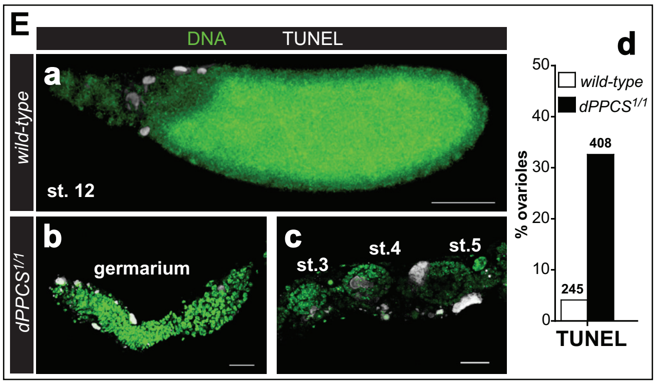


Table S1. *dPPCS*[1] affect nurse cell chromatin morphology

| Stage 6-8 nurse cell chromatin morphology | % of total (egg chambers) | | |
|---|---|---|---|
| | *wild-type* (n=407) | *dPPCS*$^{1/1}$ (n=490) | *P[dPPCS];dPPCS*$^{1/1}$ (n=307) |
| normal chromatin | 97.1 | 34.2 | 86.3 |
| aberrant chromatin | 0.5 | 11.8 | 2.0 |
| degenerative chromatin | 0.7 | 12.4 | 0.7 |
| > 15 nurse cell nuclei | 1.7 | 41.6 | 11.1 |

Nurse cells were stained with DAPI to detect the DNA. *P[dPPCS]* is a FLAG-tagged dPPCS cDNA under control of an ubiquitin promotor.

**Figure S1. *dPPCS*[1] affects germ line cell and follicle cell integrity during early oogenesis** Early oogenesis was investigated in *dPPCS*$^{1/1}$ ovaries and compared to wt. Antibodies against pH3Ser10 were used to detect mitotic chromatin. Several antibodies were used to visualize various structures and cell types. Anti-FasIII antibodies were used because FasIII is expressed in all undifferentiated follicle cells in the germarium and FasIII localization marks the polar follicle cells after the egg chambers bud from the germarium. Antibodies against

Armadillo (Arm) and DE-cadherin (DE-cad) were used because Arm and DE-cad are expressed in the adhesive junctions of the migrating follicle cells that assist in the rearrangement of the germ line cells. Antibodies against Orb were used because Orb marks oocytes. Antibodies against Vasa were used because Vasa specifically accumulates inside germ line cells. DAPI was used to stain DNA, and rhodamin-phalloidin was used to visualize F-actin. **(A)** *dPPCS* is required for nurse cell chromatin condensation, egg chamber packaging and polarity. **(Aa)** In wt egg chambers, Orb labeling reveals the wild type localization pattern of the oocytes (arrowheads). **(Ab)** *dPPCS$^{1/1}$* ovarioles contain egg chambers with multiple (arrows) or mispositioned (arrowhead) oocytes. **(Ac)** Overexpression of a FLAG-tagged dPPCS cDNA (*P[dPPCS]*) construct suppressed the occurrence of multiple oocytes and egg chambers with supernumerary nurse cells (see also **Table S1**). **(Ad)** During wt oogenesis, follicle cells are mitotically (pH3Ser10 staining) active until stage 6 and subsequently proceed into endocycling [24]. At stage 5, the nurse cell chromatin has a characteristic 5-lobed appearance and at stage 6, nurse cell chromatin is completely dispersed [25,26]. Nurse cell chromatin dispersion coincides with the mitotic-to-endocycle switch (stages 6-7), during which the follicle cells of the follicular epithelium stop mitosis and initiate endoreplication. **(Ae)** The mitotic-to-endocycle switch is intact in *dPPCS$^{1/1}$* egg chambers, and no mitotically active follicle cells are observed after stage 6. **(Ae-g)** Examples of egg chambers in which chromosomes failed to disperse properly and in which some nuclei remained 5-lobed (arrowheads). **(Ah)** Example of a stage 10 *dPPCS$^{1/1}$* egg chamber showing 2 nurse cell nuclei that are poorly replicated and small in size. **(Ai)** Example of a *dPPCS$^{1/1}$* egg chamber with supernumerary nurse cell nuclei that are heterogenous in size. **(Aj)** Example of *dPPCS* mutant egg chambers undergoing premature apoptosis as indicated by the presence of fragmented nuclei (arrowhead). **(Ak)** Example of *dPPCS$^{1/1}$* egg chambers showing a degenerative appearance. Scale bars: 150 µm (a-c), 50 µm (d-h). (B) Follicle cell migration and organization is disrupted in *dPPCS$^{1/1}$* germaria. **(Ba)** Wt cysts (marked by asterisk) in region 2b adopt a lens-shape appearance (arrow marks the stalk cells). **(Bb)** In *dPPCS$^{1/1}$* germaria, the cysts (marked by asterisk) do not adopt the characteristic lens-shape appearance. Formation of the interfollicular stalk (arrow) is severely disrupted and newly formed egg chambers display features of fusion (arrowhead). **(Bc)** Wt follicle cells that migrate between the cysts express FasIII. When the egg chambers bud from the germarium, only the polar follicle cells (asterisk) express FasIII [7,11,27]. (oo = oocyte). **(Bd)** In *dPPCS$^{1/1}$* germaria, migration of the follicle cells is disrupted (arrow) and this results in packaging defects (7-8, 10-11), mispositioning of the oocytes (9,10,12), and induces formation of egg chambers without stalks (arrowhead). Numbers mark the oocytes. **(Be-e')** In wt egg chambers, Arm and DE-cad are highly expressed in the migrating follicle cells and later in the stalk cells (arrowheads) [28-30]. Asterisks mark the position of the oocytes. **(Bf-f')** Arm and DE-cad were abnormally expressed in *dPPCS$^{1/1}$* stalk cells (arrowheads). **(Be")** Wt germ line cells express the Vasa protein. The migrating follicle cells in region 2b adopt a convex lens-shape [31] (arrows). **(Bf")** *dPPCS$^{1/1}$* germ line cells accumulated normal amounts of Vasa, demonstrating that specification of the germ line cell was not affected. Migrating follicle cells do not exhibit a convex lens-shape (arrows). Scale bars: 20 µm. **(C)** *dPPCS$^{1/1}$* egg chambers exhibit features of aberrant polar follicle cell and stalk cell specification. **(Ca)** Wt egg chambers contain two groups of polar follicle cells, one at the anterior and one at the posterior. **(Cb)** A *dPPCS$^{1/1}$* egg chamber with three groups of polar follicle cells (arrows) and a mispositioned oocyte (asterisk). **(Cc)** Examples of *dPPCS$^{1/1}$* egg chambers in which the cuboidal follicle cells are maintained in an undifferentiated state (high FasIII expression). The follicular epithelium displays a discontinuous character and the nurse cell chromatin is heterogenous in size (arrows indicate polar follicle cells). **(Cd)** Wt egg chambers are connected by an interfollicular stalk (arrow). **(Ce)** Image, obtained by making one single confocal scan, showing a *dPPCS$^{1/1}$* egg chamber in which the interfollicular stalk is missing (arrow). Note that the follicular epithelium appears to be a bilayer (arrowheads). **(Cf)** Single confocal scan showing a *dPPCS$^{1/1}$* egg chamber in which the follicle cells are accumulated in a bilayer at the posterior of the oocyte (arrowheads). **(Cg)** In *dPPCS$^{1/1}$* ovaries, the interfollicular stalks are sometimes elongated and are composed of undifferentiated follicle cells (arrowhead). Arrows mark two groups of polar follicle cells in the adjacent egg chamber. Scale bars: 50 µm (c), 20 µm (a, b, d-g). **(D)** Notch localization is disrupted in *dPPCS$^{1/1}$* germaria. Formation of the stalk and the polar cells depends on a Delta-Notch signaling route that specifies the anterior polar follicle cells, which in turn induce stalk cell formation [12,32]. Abnormal follicle cell differentiation, multiple layering and aberrant packaging have been observed in Notch mutant females [7,11,12,22,33]. **(Da-a")** In wt germaria, Notch is highly expressed in region 2b, while Notch localizes cortically in newly produced egg chambers [33]. **(Db-b")** In *dPPCS$^{1/1}$* germaria, protein levels of Notch were frequently lower/abnormal (40%; n=20 germaria) compared

with wt germaria (arrowheads). In a fused egg chamber, Notch localization at the cortical membrane was not disrupted. At the posterior half of this egg chamber low levels of FasIII were observed, while at the anterior half high levels of FasIII were visible using antibody staining, indicating that differentiation of the cuboidal follicle cells was not affected (arrows indicate polar follicle cells) Scale bars: 20 μm. **(E)** *dPPCS$^{1/1}$* egg chambers and germaria are apoptotic. Wt and *dPPCS$^{1/1}$* ovaries were investigated for apoptosis 144 h AE. **(Ea)** Under normal physiological conditions apoptosis is initiated during cytoplasmic dumping (stage 10). During stage 10, the nurse cell nuclei of wt egg chambers were positive for TUNEL staining (no staining was observed in <stage 9 egg chambers). **(Eb-d)** The follicle cells that surround the *dPPCS$^{1/1}$* germaria and egg chambers are positive for TUNEL staining, indicating that the somatically derived follicle cells were undergoing apoptosis. Although TUNEL positive nurse cells were also observed, apoptosis was most profound in the follicle cells. **(Ed)** Quantification of the percentage of ovarioles with apoptotic egg chambers. Numbers depicted at the top of each histogram represent the number of ovarioles scored. Scale bars: 100 μm.

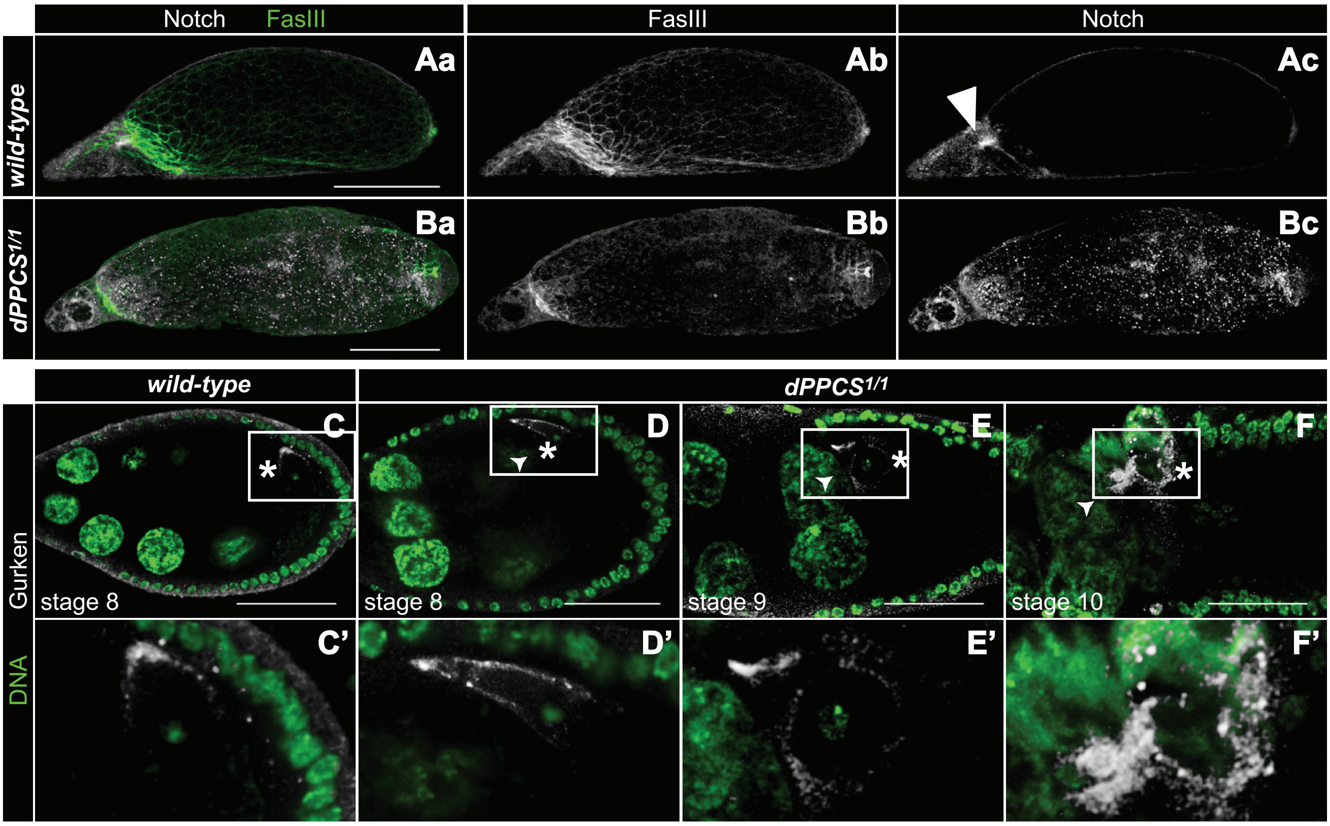


**Figure S2. Notch and Grk are abnormally localized in late stage *dPPCS$^{1/1}$* egg chambers** Wt and *dPPCS$^{1/1}$* ovaries were labeled with antibodies against Notch and Grk to investigate whether these signaling routes are affected in *dPPCS* mutant egg chambers. DAPI was used to visualize DNA. **(Aa-c)** A wt egg chamber at stage 13. The FasIII positive cells are producing the dorsal appendages and the operculum. Notch localization is restricted to the anterior follicle cells (arrowhead). **(Ba-c)** In *dPPCS* mutant stage 13 egg chambers, FasIII localization was abnormal, dorsal appendage and operculum formation was disrupted, and Notch was localized throughout the entire follicular epithelium. **(C-F)** Images obtained with single confocal scans of wt **(C)** and *dPPCS$^{1/1}$* (D-F) egg chambers. **(C)** The Grk protein localizes at the dorsoanterior corner of the oocyte in wt stage 8-9 egg chambers. **(D-F)** Grk localization was present in *dPPCS$^{1/1}$* mutant egg chambers, but the protein was frequently abnormally localized along the dorsoanterior corner in stages 8-9, most likely due to the disrupted shape of this corner **(D)** and progressively worsened when egg chambers proceeded into late stage oogenesis (stages 10-11) **(E-F)**. The nurse cell nuclei are in close proximity to the dorsoanterior corner, indicating that these nuclei were not properly anchored during dumping (arrowheads). Note that the follicular epithelium appears disorganized. Asterisks mark the position of the oocyte. Scale bars: 150 μm (A-B), 50 μm (C-F).

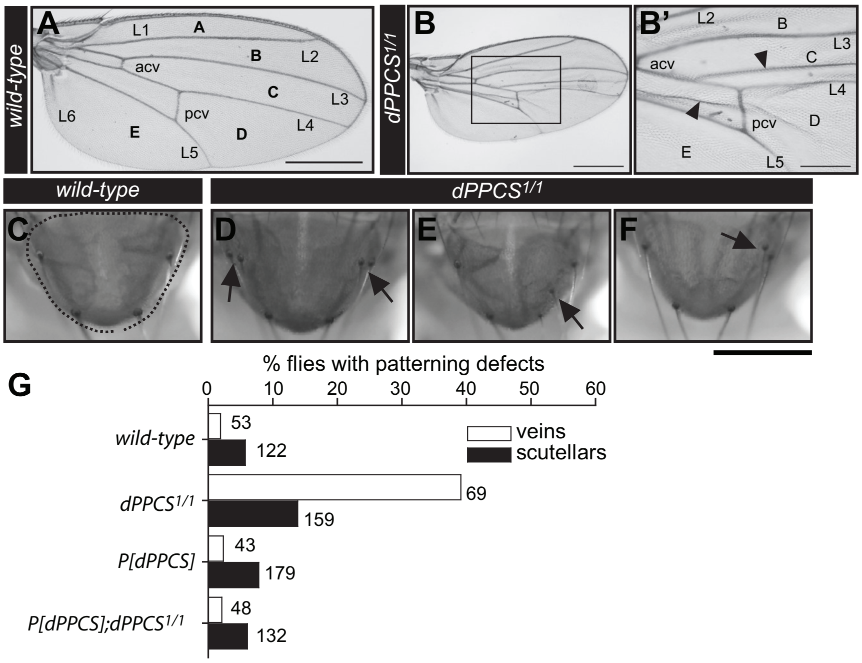


**Figure S3. Mutations in de novo CoA biosynthesis affect wing vein and scutellar patterning** Wing vein and scutellar patterning was analyzed in *dPPCS$^{1/1}$* and wt. **(A)** Dorsal wing surface of wt. (L1-L6: longitudinal veins 1 to 6; acv: anterior cross veins; and pcv: posterior cross veins) A-E (intervein sectors). **(B-B')** *dPPCS$^{1/1}$* wings displayed ectopic veins between longitudinal veins L3-L4 and L4-L5 (arrowheads). **(C)** Thorax of a wt fly. The scutellum (dashed) has a pattern of four scutellars (bristles, scutellars). **(D-F)** Thorax of *dPPCS* mutant flies developed ectopic scutellars (arrows). (G) Quantification of wing and scutellar abnormalities. Numbers represent the number of flies investigated. Scale bars: 250 µm (A-B), 100 µm (B'), 500 µm (C-F).

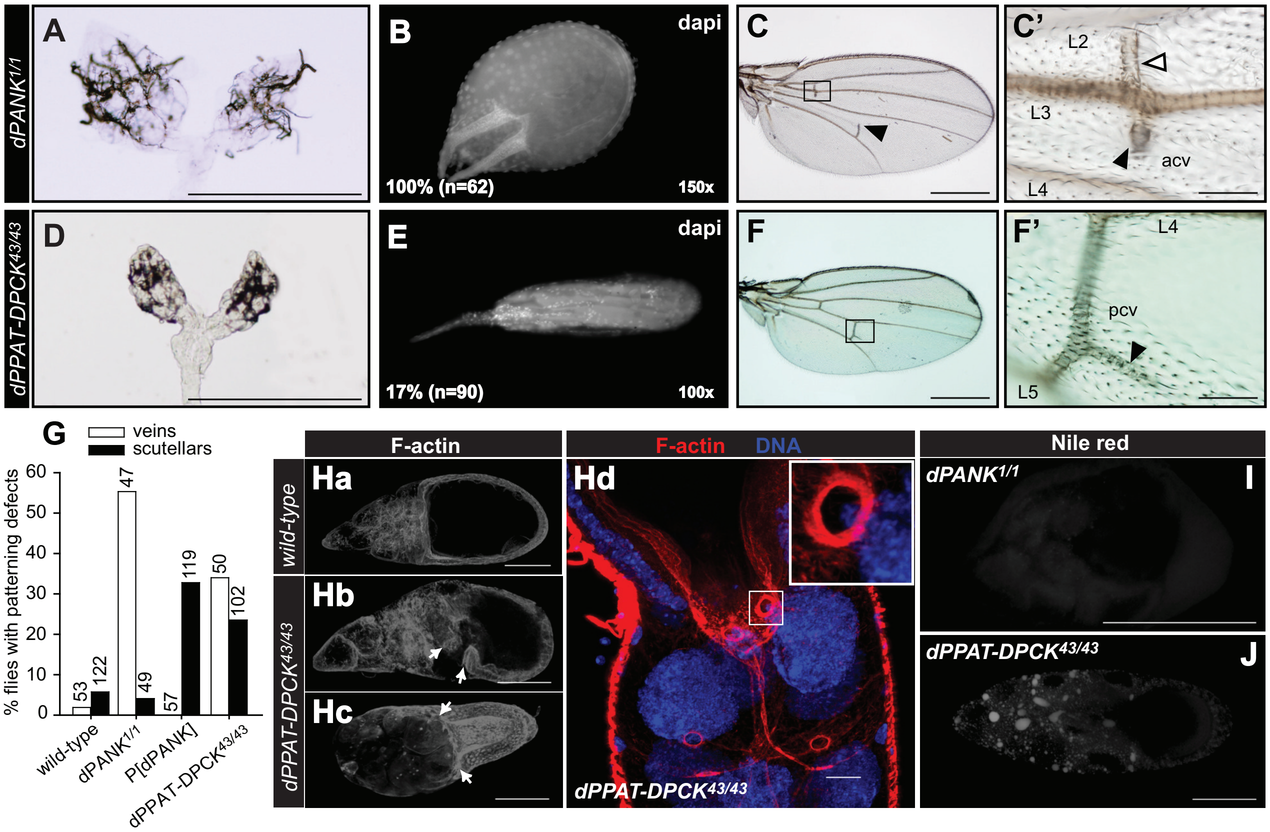

**Figure S4. Mutations in the de novo CoA biosynthesis route affect morphogenesis** Like *dPPCS*$^{1/1}$ females, *dPANK* and *dPPAT-DPCK* (NB: in Drosophila the *dPPAT* and *dDPCK* enzymes are encoded by one gene and translated into one bifunctional enzyme) mutant females have fertility defects. The *dPANK*$^{1}$, *P[dPANK]* and *dPPAT-DPCK*$^{43}$ lines have been previously described [1]. Females that carry a mutation in the *dPANK* gene did not deposit eggs, while the *dPPAT-DPCK*$^{43/43}$ females deposited 0.36 ± 0.04 eggs/24 h, of which 20.1% (n=232) were able to hatch. **(A)** *dPANK*$^{1/1}$ ovaries dissected 48 h AE are poorly developed and do not contain eggs. **(B)** Five-d-old *dPANK*$^{1/1}$ ovaries contained eggs, which are all small, ball-shaped and contain short dorsal appendages. This phenotype is identical to the eggs found in *myospheroid* [34], *Dlar* [35,36], *kugelei* [37], *dystroglycan* [38,39] and *quail* [40]. All of these genes encode proteins that are required for proper F-actin dynamics. Small ball-shaped eggs are typically due to loss of actin regulatory elements that control the polarized arrangement of F-actin fibers at the basal cortex of all follicle cells. During stages 5-8, these F-actin arrays are arranged in such a way that the fibers run perpendicular to the anteroposterior axis of the egg chamber and give the egg chamber a planar polarity that is required to create elongated eggs [23,41,42]. **(C-C')** In *dPANK*$^{1/1}$ wings, anterior cross vein (acv) and posterior cross vein (pcv) formation was incomplete (arrowheads) and mispositioned cross veins between L2 and L3 were found (boxed arrowhead). **(D)** *dPPAT-DPCK*$^{43/43}$ ovaries dissected 48 h AE are poorly developed and do not contain eggs. **(E)** 17% of the eggs from 5-d-old *dPPAT-DPCK*$^{43/43}$ females were elongated along the anteroposterior axis and exhibited a collapsed phenotype. **(F-F')** In *dPPAT-DPCK*$^{43/43}$ wings, ectopic vein formation initiated from the posterior cross vein (arrowhead). **(G)** Quantification of wing and scutellar abnormalities in *dPANK*$^{1/1}$ and *dPPAT-DPCK*$^{43/43}$ flies. Numbers represent the number of flies investigated. *dPANK*$^{1/1}$ flies did not develop ectopic macrochaetae; however, in flies that carried a FLAG-tagged dPANK cDNA under the control of an ubiquitin promoter (*P[dPANK]*) [1], an increase in the formation of macrochaetae was found, suggesting that dPANK overexpression induced the formation of ectopic scutellars. **(H)** Wt (Ha) and *dPPAT-DPCK*$^{43/43}$ (Hb-Hd) ovaries were stained with rhodamin-phalloidin to visualize the F-actin network during cytoplasmic dumping. DAPI was used to visualize DNA. **(Hb-Hc)** Cytoplasmic dumping and centripetal migration of the follicle cells (arrows) was frequently severely disrupted in *dPPAT-DPCK*$^{43/43}$ egg chambers. **(Hd)** Likely as a result of aberrant F-actin assembly, we frequently found ring canals plugged with nurse cell nuclei in dPPAT-DPCK mutant egg chambers, suggesting that aberrant dumping underlies the production of long elongated eggs as observed in E. **(I)** Production of neutral lipids (Nile red staining) was hardly detected in *dPANK*$^{1/1}$ egg chambers. (J) Production of neutral lipids (Nile red staining) and transport of lipid droplets to the oocyte was disrupted during oogenesis in *dPPAT-DPCK* mutants. Although levels of neutral lipids were not severely affected in *dPPAT-DPCK*$^{43/43}$ mutants, abnormal large lipid droplets were observed, indicating that lipid droplet formation was impaired [43] (compare **Fig. 3A**). Scale bars: 500 µm (A,D), 250 µm (C, F), 50 µm (C', F'), 100 µm (Ha-Hc, I, J), 20 µm (Hd)
